# Inhibition of AI-2 Quorum Sensing and Biofilm Formation in *Campylobacter jejuni* by Decanoic and Lauric Acids

**DOI:** 10.3389/fmicb.2021.811506

**Published:** 2022-01-13

**Authors:** Shenmiao Li, Kelvin Ka-wan Chan, Marti Z. Hua, Greta Gölz, Xiaonan Lu

**Affiliations:** ^1^Food, Nutrition and Health Program, Faculty of Land and Food Systems, The University of British Columbia, Vancouver, BC, Canada; ^2^Department of Food Science and Agricultural Chemistry, Faculty of Agricultural and Environmental Sciences, McGill University, Montreal, QC, Canada; ^3^Institute of Food Safety and Food Hygiene, Freie Universität Berlin, Berlin, Germany

**Keywords:** *Campylobacter jejuni*, quorum sensing (QS), biofilm, motility, fatty acids

## Abstract

*Campylobacter jejuni* is a major bacterial cause of human diarrheal diseases worldwide. Despite its sensitivity to environmental stresses, *C. jejuni* ubiquitously distributes throughout poultry production chains. Biofilm formation mediated by quorum sensing is suggested to be critical to the survival of *C. jejuni* in agroecosystem. *C. jejuni* possesses LuxS, the enzyme involved in the production of autoinducer-2 (AI-2) signaling molecules. In this study, two fatty acids, namely decanoic acid and lauric acid, were identified to be effective in inhibiting AI-2 activity of *C. jejuni*. Both decanoic acid and lauric acid at 100 ppm inhibited ∼90% AI-2 activity (*P* < 0.05) of *C. jejuni* without bacterial inactivation. The biofilm biomass of two *C. jejuni* strains was reduced by 10–50% (*P* < 0.05) after treatment by both fatty acids, while increased biofilm formation was observed for one *C. jejuni* strain. In addition, both fatty acids effectively reduced the motility of all tested *C. jejuni* strains. These findings can aid in developing alternative *C. jejuni* control strategies in agri-food and clinical settings.

## Introduction

*Campylobacter* is recognized as a major bacterial cause of human diarrheal diseases worldwide (Kaakoush et al., 2015). In 2010, *Campylobacter* infections and the post-infectious sequelae caused an estimated 166 million diarrheal illnesses and 37,600 deaths worldwide, resulting in significant socio-economic implications (Kirk et al., 2015). Epidemiological evidence collected from FoodNet indicated that *Campylobacter* outnumbers *Salmonella* to become the most common bacterial agent causing human diarrheal disease since 2017 in the United States (Marder et al., 2017). Moreover, the incidence of *Campylobacter* infections increased by 13% in 2019 compared to 2016–2018 (Tack et al., 2020). Among the identified species, *Campylobacter jejuni* is the predominant cause of human infections (World Health Organization, 2013). *C. jejuni* is considered a commensal bacterium in poultry that inhabits the intestine and can be spread to agri-food related environments at different segments, such as chicken farms, slaughterhouses, and processing plants (Sahin et al., 2015). Although *C. jejuni* is nutritionally fastidious and sensitive to food production-associated stresses, its adaptability allows it to survive and remain infectious in various environmental conditions (García-Sánchez et al., 2018). The most common transmission of *C. jejuni* to humans is attributed to handling and consuming contaminated animal products (Wagenaar et al., 2013).

*C. jejuni* continues to pose a significant burden to public health while tremendous efforts have been put to reduce *Campylobacter*-associated foodborne illnesses. In the early decades, antibiotics (e.g., fluoroquinolone and macrolide) were widely used as therapeutic or prophylactic drugs in human medicine and poultry production (Tang et al., 2017). Antibiotic feed additives is a cost-effective solution to increase poultry production by improving growth performance and suppressing bacterial infectious in a short time (M’Sadeq et al., 2015). Nevertheless, the emergence of antibiotic-resistant *C. jejuni* has been observed following the extensive use of antibiotics in broiler breeder farms (Guo et al., 2018; Mehdi et al., 2018). These antibiotic-resistant *C. jejuni* strains threaten the effectiveness of those commonly used antibiotics in treating human infections (Centers for Disease Control Prevention, 2019). Since an EU-wide ban on antimicrobial growth promoters in 2006, many other countries have phased out medically important antibiotic use in animal agriculture (Martin et al., 2015). In addition, stricter regulations of *Campylobacter* in poultry production have been implemented to control the incidence of *C. jejuni* infections. In 2016, the US Department of Agriculture’s Food Safety and Inspection Service updated food safety standards with lower tolerance of *Campylobacter* in raw poultry products so as to reduce campylobacteriosis (Us Department of Agriculture Food Safety and Inspection Service, 2016). Therefore, developing effective alternative control measures to reduce the prevalence of *C. jejuni* in the agroecosystem is highly necessary.

The intercellular communication system of bacteria, namely quorum sensing, is a potential target for pathogen control. Quorum sensing enables bacteria to coordinate their physiological functions to adapt to population changes, collectively act as a group, and mediates behaviors including expression of virulence factors, change of nutrient acquisition, production of public goods, and biofilm formation (Papenfort and Bassler, 2016). To orchestrate population-wide behaviors, bacteria communicate via different types of self-produced chemical signals called autoinducers. Most of the identified autoinducers in the early studies can be classified into three categories, namely *N*-acyl-homoserine lactones (AHLs), autoinducing peptides (AIPs), and autoinducer-2 (AI-2). AHLs, AIPs, are used by Gram-negative bacteria, Gram-positive bacteria, respectively (Reading and Sperandio, 2006). Besides, AI-2 as a type of universal signaling molecules are utilized by both Gram-negative and Gram-positive bacteria (Reading and Sperandio, 2006). Through quorum sensing, bacteria can take a census of surrounding cells, recognize if they are kin or not, and respond to the signals in a coordinated fashion contingent on the type and concentration of quorum sensing molecules (Papenfort and Bassler, 2016). Many studies demonstrated the great potential of quorum sensing inhibitors in controlling bacterial infections (Brackman et al., 2011; Jiang and Li, 2013; Jiang et al., 2019). By preventing the phenotypes that enhance adaption, survival, and virulence, quorum sensing inhibitors increase pathogens’ susceptibility to antimicrobials and physical treatments and pathogenicity (Bettenworth et al., 2019). In *C. jejuni*, cell density-dependent phenotypes (e.g., motility, host colonization, virulence, and biofilm formation) are associated with AI-2-mediated quorum sensing system (Plummer, 2012). S-ribosylhomocysteinase, also known as LuxS, produces AI-2 as a by-product during methionine recycling (Schauder et al., 2001), and LuxS coding gene has been identified in *C. jejuni* (Parkhill et al., 2000). AI-2 production in *luxS*-deficient *C. jejuni* mutant is completely repressed (Plummer et al., 2011). With the abolished AI-2 activity, *luxS*-deficient *C. jejuni* also showed weakened cooperative behaviors, including autoagglutination (Jeon et al., 2003), motility (Plummer et al., 2011), host colonization and biofilm formation (Quiñones et al., 2009), all of which are key factors affecting the survival of *C. jejuni*. Therefore, inhibiting quorum sensing in *C. jejuni* could be a novel approach to control this microbe in agri-food and clinical settings.

Bactericidal effects of a wide variety of phytochemicals and fatty acids, sterols, and glycerols (e.g., cinnamaldehyde, myristic acid, β-sitosterol, monomyristin, etc.) have been widely studied due to their low cost and high safety (Barbieri et al., 2017). Some short-chain and medium-chain fatty acids can be used as sanitizers for food contact surfaces with pH < 4.0 (Marriott et al., 2006). Nevertheless, carboxylic acid at high concentration and low pH are corrosive to equipment, and long-term use of acidic solution may create niches for biofilm development due to equipment tear and wear (Schmidt, 1997). A few studies reported potential AI-2 inhibitory effects of long-chain fatty acids, including linoleic acid, oleic acid, palmitic acid, and stearic acid (Widmer et al., 2007). However, the potential of quorum sensing inhibition by fatty acids at low concentrations without bactericidal effect has not been investigated yet. In this study, 12 natural-origin compounds on *C. jejuni* AI-2 activity were investigated, the chemical structure and name of each compound were listed in Supplementary Table 1. Two medium-chain fatty acids were identified to be effective on quenching AI-2 of *C. jejuni* at their subinhibitory concentration. Their effects on biofilm formation and motility were also evaluated.

## Materials and Methods

### Chemicals and Bacterial Strains

Hexanoic acid (> 98% purity), octanoic acid (> 98% purity), and decanoic acid (> 99% purity) were purchased from Alfa Aesar (Ward Hill, MA, United States). Lauric acid was obtained from Acros Organics (Fair Lawn, NJ, United States). 4-Hydroxy-2,5-dimethyl-3(2H)-furanone (≥ 98% purity), 5-Ethyl-4-hydroxy-2-methyl-3(2H)-furanone (≥ 96% purity), 2,5-Dimethyl-3-oxo-(2H)-fur-4-yl butyrate (≥ 93% purity), crotonic acid (≥ 98% purity), trans-ferulic acid (≥ 99% purity), quercetin (≥ 95% purity), trans-cinnamaldehyde (≥ 99% purity), and naringenin (≥ 95% purity) were obtained from Sigma-Aldrich (Oakville, ON, Canada). The stock solutions of hexanoic acid, octanoic acid, decanoic acid, lauric acid, 4-hydroxy-2,5-dimethyl-3(2H)-furanone, 5-ethyl-4-hydroxy-2-methyl-2(2H)-furanone, 2,5-dimethyl-3-oxo-2(H)-fur-4-yl butyrate, crotonic acid, and trans-ferulic acid were prepared by separately dissolving each compound in absolute ethanol to achieve a concentration of 20,000 ppm. The stock solution of each compound was further diluted to working solutions as 400, 300, 200, 150, 100, 50, and 25 ppm with MH broth upon the test. The stock solutions of quercetin, trans-cinnamaldehyde, and naringenin were prepared by separately dissolving each compound in absolute ethanol to achieve a concentration of 5,000 ppm. The stock solution of each compound was further diluted to working solutions as 100, 50, 25, 12.5, 6.25, and 3.125 ppm with MH broth upon the test. MH broth with 1 and 0.5% ethanol were used as the control groups. We verified that the addition of ethanol at a concentration of 1% or below did not affect *C. jejuni* cell viability and growth (data not shown).

Three *C. jejuni* strains, namely F38011 (human clinical isolate), NCTC11168 (human clinical isolate), and ATCC33560 (bovine feces isolate), were routinely cultivated either on Mueller Hinton II agar (BD BBL*™*) plates supplemented with 5% defibrinated sheep blood (MHB agar) or in MH broth (BD Difco*™*) at 37°C under microaerobic condition (85% N_2_, 10% CO_2_, 5% O_2_) with constant shaking at 175 rpm. *Vibrio harveyi* strains BB152 (AI-2 positive control) and BB170 (AI-2 reporter) were cultivated either on Marine agar 2216 (BD Difco*™*) plates or in autoinducer bioassay (AB) medium (Greenberg et al., 1979) prepared in the lab that contains 0.33 mol⋅L^–1^ NaCl, 0.05 mol⋅L^–1^ MgSO_4_, 0.2% (w/v) vitamin-free casamino acids, 1% (v/v) glycerol, 1 mmol⋅L^–1^ L-arginine (filtration sterilized), and 10 mmol⋅l^–1^ potassium phosphate (pH 7.0) at 30°C under aerobic condition with constant shaking at 175 rpm.

### Determination of Subinhibitory Concentrations

The highest subinhibitory concentration of all the selected compounds was determined using a microtiter broth dilution method, as described in a previous study (Duarte et al., 2016). In brief, overnight *C. jejuni* culture of each strain was diluted to ∼10^8^ CFU/mL in MH broth. One hundred microliters of the working solution of each compound were separately added to each well of a sterile 96-well polystyrene plate (Corning, untreated, flat-bottom), followed by inoculation of 100 μL of *C. jejuni* culture (∼10^8^ CFU/mL). Each strain that cultivated in MH broth without addition of each compound served as positive control. Blank controls were performed using sterile MH broth supplemented with the working solution of each compound at the highest concentration. Then, the inoculated 96-well plates were covered by low evaporation lids and incubated at 37°C under microaerobic condition for 48 h. The highest concentration of the compound that did not inhibit the visible growth of *C. jejuni* culture (compared to positive control) was taken as the subinhibitory concentration for the strain.

### Screening of AI-2 Inhibitors

#### Preparation of Cell-Free Supernatants

Quantification of AI-2 activity was performed via *V. harveyi* AI-2 assay was conducted following the protocol described in a previous study with minor modifications (Bassler et al., 1997). The preliminary data showed that *C. jejuni* cultivated in a static condition in a 96-well plate has a higher AI-2 activity at 48 h than that either at 24 or 72 h (Supplementary Figure 1). In addition, *C. jejuni* ATCC 33560 forms relatively less biofilm than other strains. A larger portion of AI-2 molecules was identified in biofilm supernatant than biofilm of ATCC 33560 (Supplementary Figure 2). Based on these preliminary results, thereby we selected *C. jejuni* ATCC 33560 as the representative strain and 48 h as the treatment time for AI-2 inhibitor screening.

Quorum sensing inhibition capabilities of 12 compounds at the subinhibitory concentrations against *C. jejuni* ATCC 33560 cells were evaluated at the concentration summarized in Table 1. One hundred microliters of the working solution of each compound were added to each well of sterile 96-well polystyrene plates, followed by inoculation of 100 μL of *C. jejuni* ATCC 33560 overnight culture (∼10^8^ CFU/mL). The treatment solutions were replaced with sterile MH broth and MH broth with 1% ethanol for the broth control and ethanol control. Then, the microtiter plates were covered with low evaporation lids and incubated at 37°C under the microaerobic condition for 48 h. The supernatant from each well was collected separately after incubation and then centrifuged at 8,000 × *g* for 10 min and passed through 0.22-μm PES filters. The collected cell-free supernatants (CFSs) were stored at –20°C until further usage.

**TABLE 1:** Concentration of each compound used in the screening of quorum sensing inhibitory effects.

Compound	Concentration (ppm)
Hexanoic acid	200
Octanoic acid	100
Decanoic acid	100
Lauric acid	100
4-Hydroxy-2,5-dimethyl-3(2H)-furanone	200
5-Ethyl-4-hydroxy-2-methyl-2(2H)-furanone	200
2,5-Dimethyl-3-oxo-2(H)-fur-4-yl butyrate	200
Crotonic acid	200
Trans-Ferulic acid	200
Quercetin	25
Trans-cinnamaldehyde	12.5
Naringenin	25

#### *Vibrio harveyi* Autoinducer-2 Bioassay

To detect the AI-2 activity in each collected CFSs collected in section “Preparation of Cell-Free Supernatants.” The reporter strain *V. harveyi* BB170 and the positive control strain *V. harveyi* BB152 were grown in AB medium for 16 h at 30°C under the aerobic condition with constant shaking at 175 rpm. The *V. harveyi* BB170 was then diluted 5,000-fold with fresh AB medium. The CFSs *V. harveyi* BB152 were obtained by centrifuging the culture supernatant at 8,000 × *g* for 10 min and passed through 0.22-μm PES filters. One hundred and eighty microliters of diluted *V. harveyi* BB170 culture were added to each well of a sterile white 96-well plate (opaque, flat bottom). Twenty microliters of collected *C. jejuni* and *V. harveyi* BB152 CFSs were separately added to each well containing diluted reporter suspension. One hundred and eighty microliters of diluted reporter suspensions with 20 μL of uninoculated AB medium were served as the negative control. In addition, 20 μL of MH broth was served as broth control, [Frame1]and 20 μL of MH broth containing 0.5% ethanol was used as ethanol control. The 96-well plate was covered by a low evaporation lid and incubated at 30°C with aeration (169 rpm). The bioluminescence signal of each well was measured after 4.5 h incubation using a Tecan microplate reader (Infinite 200 Pro; Tecan Life Sciences). The bioluminescence signals of ethanol control were subtracted from the treatment groups as baselines, and the signals of broth control were subtracted from non-treated groups.

### Biofilm Cultivation

*C. jejuni* biofilm was cultivated according to the protocol described in a previous study with some modifications (Müsken et al., 2010). Three *C. jejuni* strains were separately cultivated in MH broth for 16 h and then diluted to ∼2 × 10^8^ CFU/mL. One hundred microliters of *C. jejuni* culture were added to each well of a sterile polystyrene 96-well plate, followed by the addition of 100 μL of decanoic acid and lauric acid working solutions, respectively. MH broth and MH broth with 0.5% ethanol were separately served as broth control and ethanol control. The plates were covered with low evaporation lids and incubated at 37°C under the microaerobic condition for 72 h.

### Quorum Sensing Inhibitory Effect

#### Effect of Fatty Acids on *Campylobacter jejuni* AI-2 Activity During Biofilm Formation

To determine the quorum sensing inhibitory effect of both decanoic acid and lauric acid, the supernatant in each well of the 96-well plate for biofilm cultivation was separately collected after 72-h incubation. The collected supernatant was centrifuged at 8,000 × *g* for 10 min and passed through 0.22-μm PES filters. CFSs were stored at –20°C until further use.

The AI-2 activity in the supernatant during biofilm formation was then determined using *V. harveyi* AI-2 assay as aforementioned in 2.3. In addition, MH broth (20 μL) was served as broth control, and MH broth containing 0.5% ethanol (20 μL) was used as ethanol control. The signals of broth control were subtracted from non-treated groups, and the bioluminescence signals of ethanol control were subtracted from the treatment groups as baselines.

#### Effect of Fatty Acid on AI-2 Activity of *Campylobacter jejuni* Cell-Free Supernatants

To determine whether there is a direct interaction between *C. jejuni* AI-2 signaling molecules and fatty acids, *C. jejuni* was grown in MH broth overnight at 37°C under the microaerobic condition. Cell-free supernatants were collected by centrifugation and filtration as aforementioned. One hundred microliters of the collected cell-free supernatants were separately mixed with 100 μL of 200, 100, and 50 ppm decanoic acid and lauric acid working solutions and incubated for 1 h at 37°C. AI-2 activity of each treated cell-free supernatant was determined using *V. harveyi* AI-2 assay.

### Quantification of *Campylobacter jejuni* Biofilm Biomass

Crystal violet staining assay was used to quantify the total biomass of formed *C. jejuni* biofilms according to the protocol described in a previous study with modifications (Feng et al., 2018). Each well of the polystyrene plate was washed twice using PBS and air-dried for 30 min. One hundred and fifty microliters of 1% (w/v) crystal violet solution was added into each well to stain the attached biofilm for 15 min. Then, the crystal violet solution was removed from each well, followed by washing three times with sterile deionized water to remove any unbounded staining residuals. The plates were air-dried for 30 min, followed by the addition of 200 μL of 95% ethanol (v/v) to dissolve the biofilm-associated crystal violet for 10 min. The absorbance level of the dissolved dye in each well was determined using a microplate reader at 595 nm (Infinite 200 Pro; Tecan Life Sciences). Sterile MH broth and MH broth with each fatty acid were stained to serve as broth control and treatment controls. The broth control and treatment controls were subtracted from the non-treated and treated groups, respectively, as the baselines.

### Bacterial Motility Assay

The effect of fatty acids on *C. jejuni* cell motility was assessed using the soft agar plate assay (Kalmokoff et al., 2006). Soft agar plates contained 25 mL of MH broth supplemented with 0.4% agar with or without the addition of decanoic acid and lauric acid. Soft agar plates that contained 0.5% ethanol were used as the negative control. *C. jejuni* overnight culture was adjusted to ∼10^8^ CFU/mL, and 2 μL of the adjusted culture was stab inoculated at the center of the soft agar plates. The inoculated plates were then incubated at 37°C for 48 h under the microaerobic condition. The motility of *C. jejuni* cells was determined by measuring the distance of bacterial migrating from the inoculation site.

### Statistical Analysis

All experiments were conducted at least in three biological replicates. Results were presented as the mean ± standard deviation. Data analysis and visualization were performed using Origin (version OriginPro 2020, OriginLab Corporation, United States). One-way ANOVA followed by appropriate *post hoc* test was used to determine if the difference was statistically significant (*P* < 0.05).

## Results and Discussion

### Subinhibitory Concentrations of Fatty Acids

Subinhibitory concentration of an antimicrobial is the concentration below the minimum inhibitory concentration of that compound. Exposing microorganisms to the subinhibitory concentration of an antimicrobial allows the observation of its effects on cellular processes without disturbing cell viability. Quorum sensing is a cell-to-cell communication process underpinned by various biological processes (Papenfort and Bassler, 2016). Using subinhibitory concentration to study quorum sensing quenching effect can largely avoid bias caused by bactericidal effect.

The highest subinhibitory concentrations of the tested compounds against four *C. jejuni* isolates are shown in Table 2. Among 12 tested compounds, hexanoic acid, 4-hydroxy-2,5-dimethyl-3(2H)-furanone, 5-ethyl-4-hydroxy-2-methyl-2(2H)-furanone, 2,5-dimethyl-3-oxo-2(H)-fur-4-yl butyrate, crotonic acid, and trans-ferulic acid did not show growth inhibitory effect on all four strains at the concentration of 200 ppm, which was the highest tested concentration. For the four fatty acids (i.e., hexanoic acid, octanoic acid, decanoic acid, and lauric acid), the inhibitory concentrations decreased along with the increase of alkyl chain length. Susceptibility of the three tested *C. jejuni* strains to octanoic acid, lauric acid, quercetin, and naringenin was slightly different. For example, *C. jejuni* ATCC 33560 was more susceptible to octanoic acid than the other tested strains.

**TABLE 2:** The highest subinhibitory concentrations of the selected compounds against three *C. jejuni* isolates.

Compound	Strain	*SIC* (ppm)
Hexanoic acid	F38011	No inhibition at conc. ≤ 200
	11168	
	33560	
Octanoic acid	F38011	No inhibition at conc. ≤ 200
	11168	
	33560	150
Decanoic acid	F38011	150
	11168	
	33560	
Lauric acid	F38011	100
	11168	
	33560	
4-Hydroxy-2,5-dimethyl-3(2H)-furanone	F38011	No inhibition at conc. ≤ 200
	11168	
	33560	
5-Ethyl-4-hydroxy-2-methyl-2(2H)-furanone	F38011	No inhibition at conc. ≤ 200
	11168	
	33560	
2,5-Dimethyl-3-oxo-2(H)-fur-4-yl butyrate	F38011	No inhibition at conc. ≤ 200
	11168	
	33560	
Crotonic acid	F38011	No inhibition at conc. ≤ 200
	11168	
	33560	
Trans-Ferulic acid	F38011	No inhibition at conc. ≤ 200
	11168	
	33560	
Quercetin	F38011	25
	11168	50
	33560	25
Trans-cinnamaldehyde	F38011	12.5
	11168	
	33560	
Naringenin	F38011	50
	11168	25
	33560	25

### Selection of AI-2 Inhibitors

**FIGURE 1:**
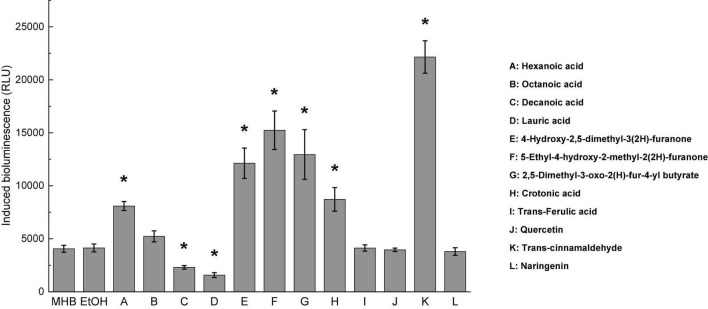
Screening of *C. jejuni* autoinducer-2 inhibitor. Bioluminescence activity of *C. jejuni* ATCC 33560 treated with 12 different compounds was compared to the ethanol control (EtOH). MH broth containing 0.5% ethanol was used as ethanol control. Each bar represents the average of three biological replicates with standard deviation. MHB was serve as broth control and EtOH was negative control. * Statistical significance between negative control and treated samples was determined by one-way ANOVA coupled with Dunnett’s multiple comparisons test (*P* < 0.05 was considered as statistically significant).

Twelve compounds were screened and assessed for their ability to inhibit AI-2 activities of *C. jejuni* ATCC 33560 at the subinhibitory concentrations, as shown in Table 1. The inhibitory effects of AI-2 mediated quorum sensing activity in *C. jejuni* by the compounds were determined as the intensity of bioluminescence signal emitted by the reporter strain *V. harveyi* BB170. Among the 12 compounds, both decanoic acid and lauric acid significantly inhibited AI-2 activities of *C. jejuni*, whereas hexanoic acid, three furanones, crotonic acid, and trans-cinnamaldehyde increased the AI-2-induced bioluminescence (Figure 1). Octanoic acid, trans-ferulic acid, quercetin, and naringenin had no significant effect on AI-2 activity of *C. jejuni* (*P* > 0.05). Therefore, decanoic acid and lauric acid might be able to quench AI-2 mediated quorum sensing activities in *C. jejuni*. In addition, there was no significant difference in bioluminescence activities between MH broth control and ethanol control (*P* > 0.05). Thus, the effect of carrier ethanol on *C. jejuni* AI-2 activity at 0.5% was negligible.

### Quorum Sensing Inhibitory Effect of Decanoic Acid and Lauric Acid

#### Quorum Sensing Inhibitory Effect During *Campylobacter jejuni* Biofilm Formation

**FIGURE 2:**
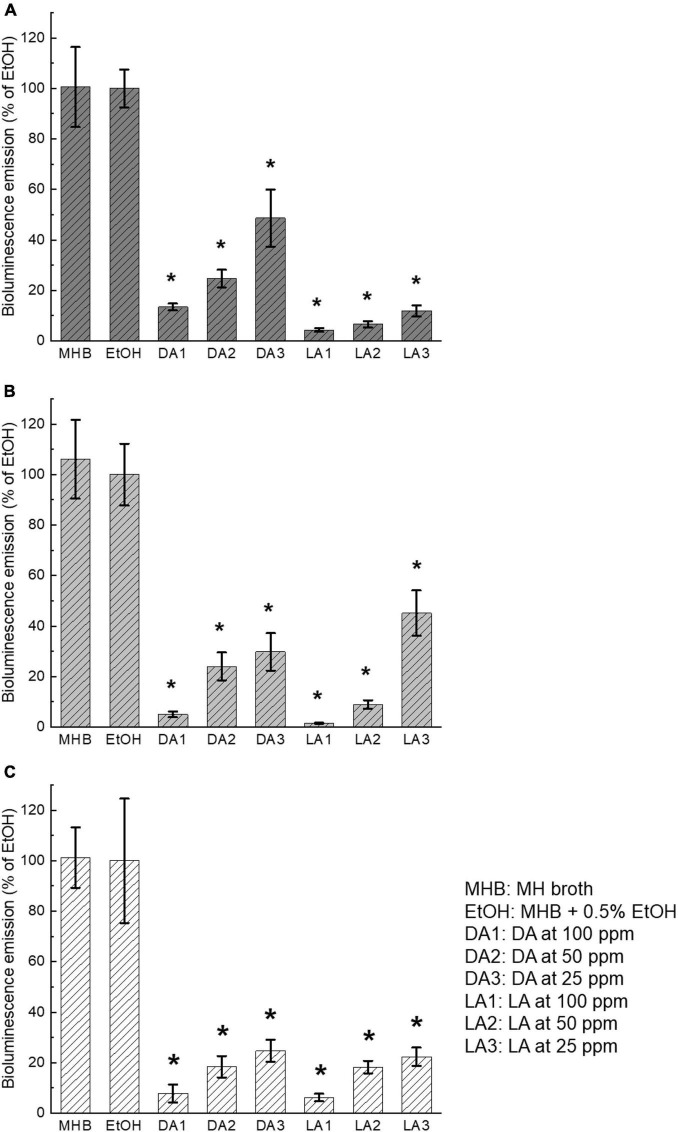
Inhibition of *C. jejuni* AI-2 activity by decanoic acid (DA) and lauric acid (LA). *C. jejuni* strain F38011 **(A)**, NCTC11168 **(B)**, ATCC33560 **(C)**. Bioluminescence emission of *V. harveyi* BB170 with the addition of cell-free supernatants collected from the supernatant of *C. jejuni* biofilm incubated with or without fatty acids for 72 h at 37°C under microaerobic condition was separately determined. MH broth containing 0.5% ethanol was used as ethanol control. MHB is the broth control and EtOH is the negative control. Bioluminescence level of the negative control was set at 100%. Bioluminescence emission of each treatment was normalized to the control. Three biological replicates were performed. * Statistical significance between negative control and treated samples was determined by one-way ANOVA coupled with Dunnett’s multiple comparisons test (*P* < 0.05 was considered statistically significant).

AI-2 activities within the supernatant of biofilms of all three *C. jejuni* strains were significantly reduced (*P* < 0.05) by both decanoic acid and lauric acid at all the tested subinhibitory concentrations (100 ppm, 50 ppm, 25 ppm) compared to the ethanol control (Figure 2). There was no significant difference in AI-2 activity between MH broth control and ethanol control, indicating that 0.5% ethanol added in the broth as the carrier solvent did not affect *C. jejuni* AI-2 activity. The addition of different concentrations of either decanoic acid or lauric acid demonstrated a concentration-dependent inhibitory effect on *C. jejuni* AI-2 activity. Lauric acid at 100 ppm showed the most substantial quorum quenching effect on all *C. jejuni* strains used in this study. However, the inhibitory effects of decanoic acid and lauric acid varied among different *C. jejuni* strains. For example, AI-2 activity of *C. jejuni* F38011 was reduced > 80% by lauric acid at all three concentrations after 3-day incubation (Figure 2A). In comparison, lauric acid at 25 ppm only decreased 65% of AI-2 activity of *C. jejuni* NCTC11168 (Figure 2B).

#### Influence of Fatty Acids on AI-2 Activity of *Campylobacter jejuni* Cell-Free Supernatants

**FIGURE 3:**
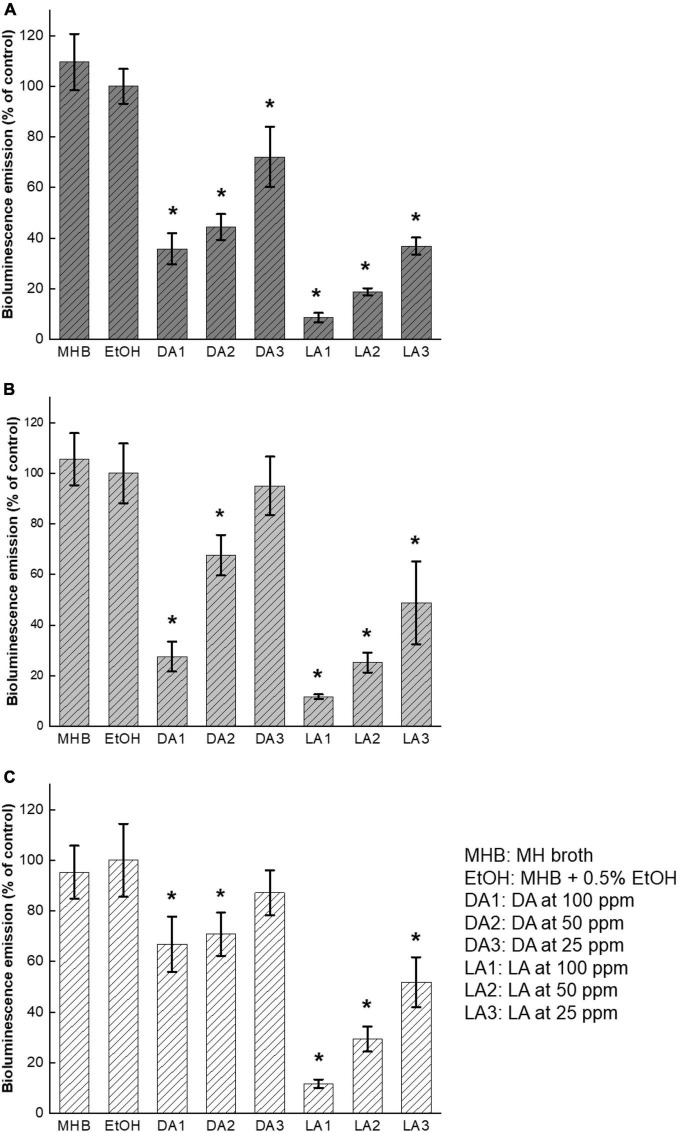
Influence of decanoic acid (DA) and lauric acid (L.A.) on AI-2 activity in *C. jejuni* cell-free supernatants. *C. jejuni* strain F38011 **(A)**, NCTC11168 **(B)**, and ATCC33560 **(C)**. Cell-free supernatant from each *C. jejuni* culture grown in MH broth was incubated with fatty acids for 1 h, respectively. MH broth containing 0.5% ethanol was used as ethanol control. MHB is the broth control and EtOH is the negative control. Bioluminescence level of the negative control was set at 100%. Bioluminescence emission of each treatment was normalized to the control. Three biological replicates were performed. * Statistical significance between negative control and treated samples was determined by one-way ANOVA coupled with Dunnett’s multiple comparisons test (*P* < 0.05 was considered statistically significant).

To investigate whether there is a direct interaction between fatty acids and *C. jejuni* AI-2 molecules, cell-free supernatants collected from *C. jejuni* overnight culture were directly incubated with decanoic acid and lauric acid at the subinhibitory concentrations, 100 ppm, 50 ppm, 25 ppm, respectively. The inhibitory effect was significant (*P* < 0.05) in all the treatment groups except for decanoic acid at 25 ppm on *C. jejuni* NCTC11168 and ATCC33560 (Figure 3). The results of direct interaction were similar to those shown in Figure 2. Concentration-dependent inhibitory effect of decanoic acid and lauric acid was observed. Lauric acid at 100 ppm showed the most potent inhibition against *C. jejuni* AI-2 activity.

Quorum sensing circuits involve a range of enzymes and receptors so that there are multiple potential targets for quorum quenching. Production of signaling molecules can be inhibited by repression/inactivation of enzymes required for A.I.s synthesis or by inactivation of the AI molecules. Besides, quorum sensing can be inhibited at the perception stage by blocking the receptors (Hentzer and Givskov, 2003). A previous study reported that several long-chain fatty acids identified from poultry meat wash were able to reduce the activity of *in vitro* synthesized AI-2. The authors speculated that the inhibition could be caused by either direct interaction with AI-2 molecules or interference of AI-2 receptor (Widmer et al., 2007). In the current study, inhibition of AI-2 activity was observed in both circumstances, namely mixing fatty acids with cell-free supernatants and treating *C. jejuni* cells. Our preliminary experiment confirmed that treatment of decanoic acid and lauric acid at 200 ppm did not affect the viability of the *V. harveyi* BB170 reporter strain. Thus, we speculated that interaction between decanoic acid and lauric acid with the signaling molecules or the receptor resulted in the loss of AI-2 activities.

### Effect of Fatty Acids on *Campylobacter jejuni* Biofilm Biomass

**FIGURE 4:**
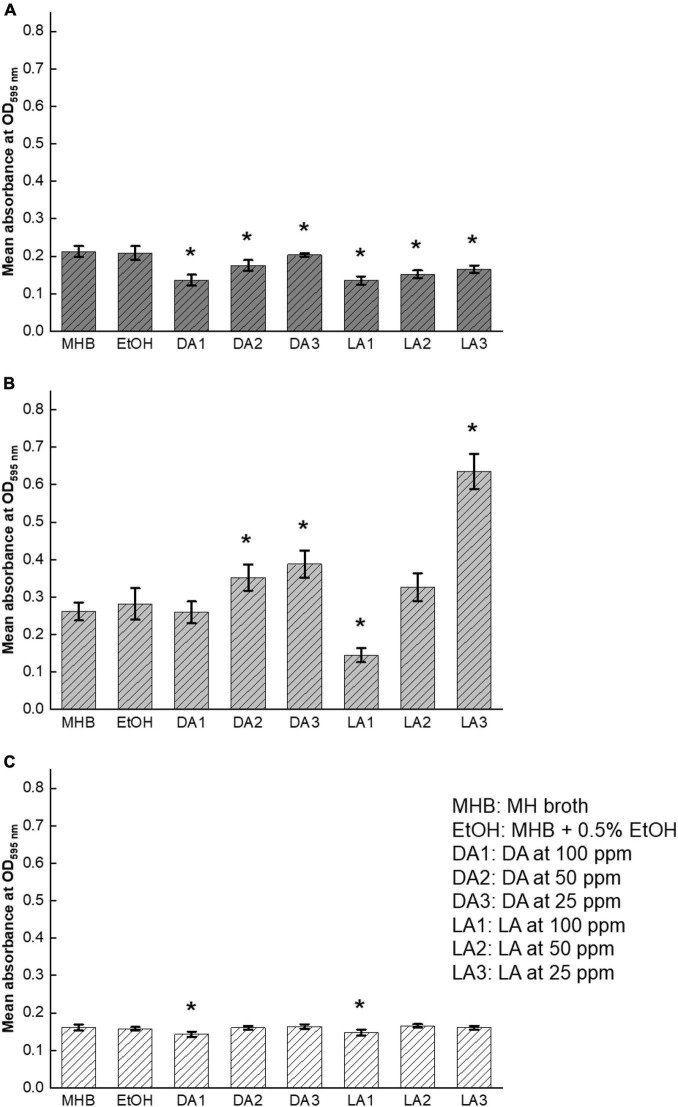
Effect of decanoic acid (DA) and lauric acid (L.A.) on biofilm formation of *C. jejuni*. *C. jejuni* strain F38011 **(A)**, NCTC11168 **(B)**, and ATCC33560 **(C)**. Total biomass of the formed biofilms was quantified by crystal violet staining at OD_595 nm_ after 72 h incubation with or without fatty acids at 37°C under microaerobic condition. MH broth containing 0.5% ethanol was used as ethanol control. MH broth and MH broth containing 0.5% EtOH are served as the broth control and negative control, respectively. Three biological replicates were performed. * Statistical significance between negative control and treated samples was determined by one-way ANOVA coupled with Dunnett’s multiple comparisons test (*P* < 0.05 was considered as statistically significant).

The effects of decanoic acid and lauric acid at different subinhibitory concentrations on *C. jejuni* biofilm formation are shown in Figure 4. There was a variation between inhibitory effects on quorum sensing and biofilm formation. Although both decanoic acid and lauric acid were potent in inhibiting AI-2 activity of *C. jejuni*, they were not effective in inhibiting *C. jejuni* biofilm formation at low concentrations. Among the three tested *C. jejuni* strains, decanoic acid at 100 ppm reduced the total biofilm biomass of *C. jejuni* F38011 and ATCC33560 by 35 and 10%, respectively (Figures 4A,C). Lauric acid at 100 ppm reduced biofilm formation of all three *C. jejuni* strains, ranging from 50% for NCTC11168 to 10% for F38011 and ATCC33560. However, a stimulating effect on *C. jejuni* NCTC11168 biofilm formation was observed at the treatment groups of 50 and 25 ppm decanoic acid and 25 ppm lauric acid.

*C. jejuni* biofilm formation has been recognized as one of AI-2 mediated bacterial social behaviors (Plummer, 2012). Reeser et al. (2007) reported that biofilm formation level was lower in *luxS*-deficient *C. jejuni* mutant than that of wildtype, and biofilm formation ability of the mutant was partially restored by adding AI-2 containing *C. jejuni* cell-free supernatants. However, another study indicated that phenotypes of *luxS-*deficient *C. jejuni* mutants varied along with the changes of mutagenesis, strains, and cultivation conditions (Adler et al., 2014). Besides, biofilm formation capabilities of various *C. jejuni* strains are different due to the variations in genetic features (Gunther and Chen, 2009). Because of the potential differences, we used three *C. jejuni* isolates from different origins to ensure the results were representative. Decanoic acid and lauric acid effectively reduced biofilm formation of *C. jejuni* F38011, but not NCTC11168, regardless of their evident inhibitory effect of AI-2 activity on this strain. However, biofilm formation is a highly complex and dynamic process. Multifaceted mechanisms, such as modulation of non-coding RNAs and transcriptional regulators, also involved in the orchestration of biofilm formation, are regulated via quorum sensing (Goo et al., 2015). Moreover, variation in growth rate of bacteria, formation of microcolonies at early biofilm formation stage, and threshold of detection of quorum sensing molecules can also lead to the difference in responding to AI-2 changes (Hense et al., 2007). Taken together, the relationship between inhibition of quorum sensing and reduction of biofilm formation is not straightforward.

### Effect of Fatty Acids on *Campylobacter jejuni* Cell Motility

**FIGURE 5:**
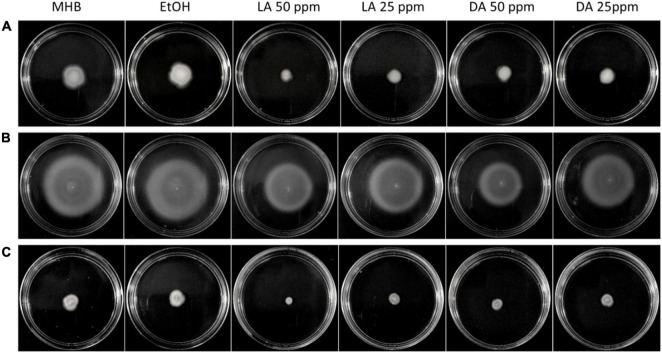
Inhibition of *C. jejuni* cell motility by lauric acid (L.A.) and decanoic acid (DA). Swarming of *C. jejuni* F38011 **(A)**, NCTC11168 **(B)**, and ATCC33560 **(C)** in soft agar (MHB + 0.4% agar) was recorded in the absence and presence of specific concentrations of fatty acids after 72 h incubation at 37°C in a microaerobic condition. Soft agar containing 0.5% EtOH is served as the negative control.

Flagella-driven motility has been validated to be one of the virulence factors of *C. jejuni* because it is critical for its colonization (Plummer, 2012). This bacterial process has been associated with AI-2 mediated quorum sensing and plays an important role in biofilm formation (Brackman et al., 2011). *C. jejuni* motility levels varied among different strains. *C. jejuni* NCTC11168 was higher motile than F38011 and ATCC33560 while *C. jejuni* NCTC11168 showed the largest migration distance (Figure 5). Interestingly, this strain also showed the highest level of biofilm formation among the three tested strains (Figure 4). *C. jejuni* motility mediated by AI-2 was associated with the development of biofilm structure and survival of *C. jejuni*. We evaluated the effects of decanoic acid and lauric acid on *C. jejuni* motility and identified that both decanoic acid and lauric acid were able to reduce the motility of all tested strains (Figure 5). Inhibition of *C. jejuni* migration was more evident at a high concentration of fatty acid treatment than those low concentration groups.

## Conclusion

Both decanoic acid and lauric acid at subinhibitory concentrations effectively reduced the AI-2 activity of *C. jejuni* by over 50% after 72-h incubation. Direct addition of either decanoic acid or lauric acid to *C. jejuni* cell-free supernatants also decreased AI-2 activity in a concentration-dependent manner. Both fatty acids reduced *C. jejuni* F38011 biofilm formation at all tested concentrations, whereas reduction of biofilm formation of *C. jejuni* ATCC33560 was only achieved by decanoic acid and lauric acid at 100 ppm. Motility of all tested *C. jejuni* strains was impaired by both fatty acids. The effect of fatty acids on *C. jejuni* biofilm formation might be determined by a complex mechanism of action. Our current results provide new insights into the use of fatty acids for *C. jejuni* control in agri-food and clinical settings.

## Data Availability Statement

The original contributions presented in the study are included in the article/Supplementary Material, further inquiries can be directed to the corresponding author/s.

## Author Contributions

SL and XL developed this project. SL and GG conceptualized and designed the experiments. SL, KC, and MH performed the experiment. SL analyzed the data. SL, MH, and XL drafted the manuscript. All authors contributed to the article and approved the submitted version.

## Conflict of Interest

The authors declare that the research was conducted in the absence of any commercial or financial relationships that could be construed as a potential conflict of interest.

## Publisher’s Note

All claims expressed in this article are solely those of the authors and do not necessarily represent those of their affiliated organizations, or those of the publisher, the editors and the reviewers. Any product that may be evaluated in this article, or claim that may be made by its manufacturer, is not guaranteed or endorsed by the publisher.
