# Supplementary material for: Inhibition of AI-2 Quorum Sensing and Biofilm Formation in Campylobacter jejuni by Decanoic and Lauric Acids
Source: Front Microbiol. 2022 Jan 13;12:811506. doi: 10.3389/fmicb.2021.811506 (PMC8793694; doi:10.3389/fmicb.2021.811506)
Supplement: Supplementary file 1 [file Data_Sheet_1.docx]

Supplementary Material

# Supplementary Tables

Supplementary Table S1 The chemical structure and molecular weight of the selected compounds.

| Compound | Chemical Structure | M.W. (g/mol) |
| --- | --- | --- |
| Hexanoic acid | 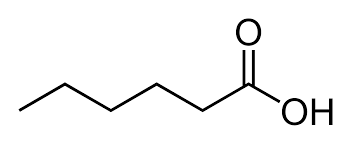 | 116.16 |
|  |  |  |
|  |  |  |
| Octanoic acid | 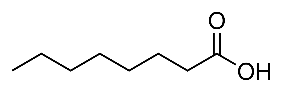 | 144.21 |
|  |  |  |
|  |  |  |
| Decanoic acid | 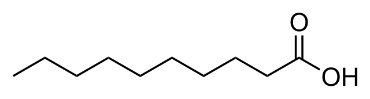 | 172.26 |
|  |  |  |
|  |  |  |
| Lauric acid | 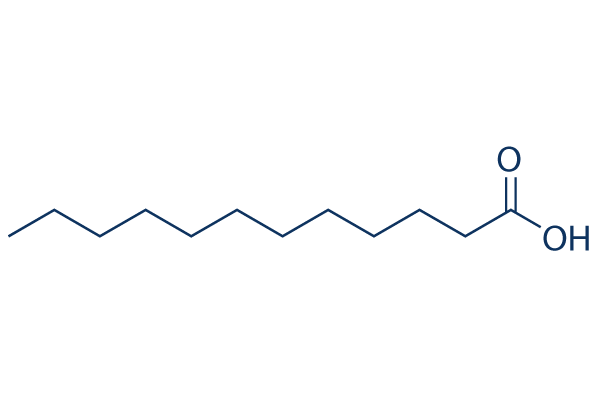 | 200.32 |
|  |  |  |
|  |  |  |
| 4-Hydroxy-2,5-dimethyl-3(2H)-furanone | 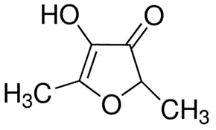 | 128.13 |
|  |  |  |
|  |  |  |
| 5-Ethyl-4-hydroxy-2-methyl-2(2H)-furanone | 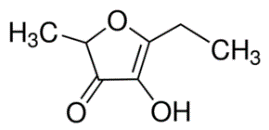 | 142.15 |
|  |  |  |
|  |  |  |
| 2,5-Dimethyl-3-oxo-2(H)-fur-4-yl butyrate | 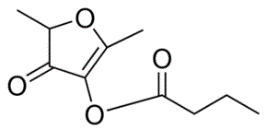 | 198.22 |
|  |  |  |
|  |  |  |
| Crotonic acid | 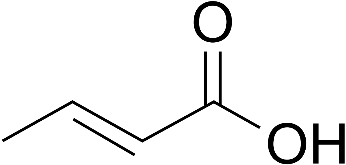 | 86.09 |
|  |  |  |
|  |  |  |
| Trans-Ferulic acid | 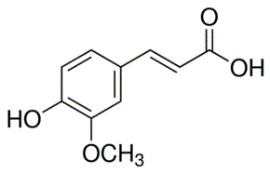 | 194.18 |
|  |  |  |
|  |  |  |
| Quercetin | 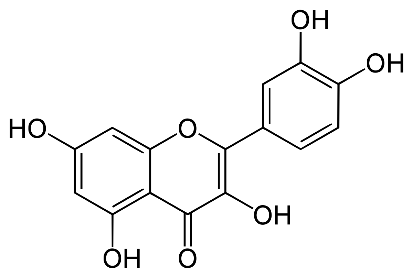 | 302.23 |
|  |  |  |
|  |  |  |
| Trans-cinnamaldehyde | 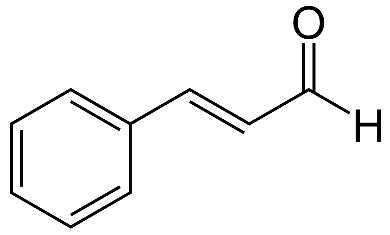 | 132.16 |
|  |  |  |
|  |  |  |
| Naringenin | 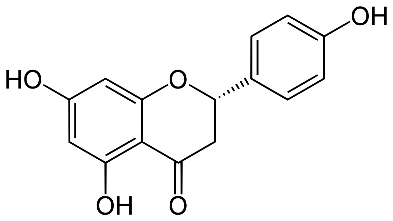 | 272.25 |
|  |  |  |
|  |  |  |

# Supplementary Figures

Supplementary Figure 1. AI-2 Activity of *C. jejuni* cell-free-supernatants (CFSs) at different incubation periods. *C. jejuni* overnight culture was adjusted to OD_600nm_ = 0.3 and further diluted to ~2 × 10^8^ CFU/mL. The time right after the dilution was taken as 0 h. Bioluminescence emission of *V. harveyi* BB170 with the addition of cell-free supernatant collected from *C. jejuni* incubated for 0, 24, 48, and 72 h at 37°C under microaerobic condition for 72 hours was measured. Measurements were performed 4.5 h after the addition of the cell-free supernatant. Three biological replicates were performed. Each bar represents the average of three biological replicates with standard deviation.

Supplementary Figure 2. Bioluminescence emission of *V. harveyi* BB170 with the addition of cell-free supernatant collected from the biofilm and supernatant phases of *C. jejuni* strains that were incubated at 37°C under microaerobic condition for 72 hours. Measurements were performed 4.5 h after the addition of the cell-free supernatant. Three biological replicates were performed. Each bar represents the average of three biological replicates with standard deviation.
